# Absolute Numerosity Discrimination as a Case Study in Comparative Vertebrate Intelligence

**DOI:** 10.3389/fpsyg.2020.01843

**Published:** 2020-08-07

**Authors:** Andreas Nieder

**Affiliations:** Animal Physiology Unit, Institute of Neurobiology, University of Tübingen, Tübingen, Germany

**Keywords:** monkey (*Macaca mulatta*), crow, number cognition, categorication, intelligence

## Abstract

The question of whether some non-human animal species are more intelligent than others is a reoccurring theme in comparative psychology. To convincingly address this question, exact comparability of behavioral methodology and data across species is required. The current article explores one of the rare cases in which three vertebrate species (humans, macaques, and crows) experienced identical experimental conditions during the investigation of a core cognitive capability – the abstract categorization of absolute numerical quantity. We found that not every vertebrate species studied in numerical cognition were able to flexibly discriminate absolute numerosity, which suggests qualitative differences in numerical intelligence are present between vertebrates. Additionally, systematic differences in numerosity judgment accuracy exist among those species that could master abstract and flexible judgments of absolute numerosity, thus arguing for quantitative differences between vertebrates. These results demonstrate that Macphail’s Null Hypotheses – which suggests that all non-human vertebrates are qualitatively and quantitatively of equal intelligence – is untenable.

## Introduction

Intelligence, broadly defined, is the general capacity to solve problems (Macphail, 1987). Whether non-human vertebrate species differ in intelligence remains hotly debated in comparative psychology. After a survey of experimental studies, Macphail (1985) adopted the “null hypothesis” and concluded that no intelligence difference, either qualitative or quantitative, had yet been demonstrated among non-human vertebrates. He argued that the alleged difference in intellect could instead be attributed to a difference in some extraneous “contextual variable,” such as species-specific variability in perception, motivation, or motor skills (Macphail, 1985, 1987).

The current article re-examines Macphail’s null hypothesis in the realm of numerical competence. Estimating numerosity, the number of items in a set, is a type of abstract categorization that is central to adaptive and intelligent behavior (Miller et al., 2003). In numerical categorization, the specific sensory features of objects or events are irrelevant since what matters is the sheer presence of elements in a set. Because humans and non-human animals share an approximate capability to estimate numerosity (Nieder, 2019) numerosity judgments offer a “window of opportunity” to gain insights into cognitive capabilities in a comparative way across phylogeny.

As pointed out by Macphail (1985, 1987) comparing the performances of different vertebrate species requires commensurable approaches and data sets in order to avoid methodological confounds. This article exploits one of the rare cases in which this requirement is fulfilled; it quantitatively explores absolute numerosity judgments that have been collected under virtually identical experimental conditions in three vertebrate species (humans, macaques, and crows). Evivalent computer-controlled visual task protocols were applied for all three species in the same laboratory environment, minimizing the variability due to task differences that usually hampers comparative behavioral research. Additionally, all three species share an acute visual sense, motivation to learn, drive to perform tasks, and comparable volitional motor dexterity (hand movements in primates, and beak/head movement in birds) that ensure analogous contextual variables. If performance differences surface under these conditions that rule out methodological and contextual variables, they can be explained by true quantitative differences in numerical capabilities as a type of intelligence. Moreover, if such absolute numerosity judgments are only mastered by certain cognitively advanced vertebrates, such as mammals and birds, it stands to reason that qualitative differences in intelligence also exist among vertebrates.

## From Relative to Absolute Numerosity Judgments

The most intensely studied form of numerical competence in animal cognition are “relative numerosity” judgments (sometimes also termed “numerousness” judgments). Here, an animal’s often spontaneous ability to select the numerical quantity that is larger relative to another quantity is tested (Nieder, 2020a). For instance, when choosing between food items (Stancher et al., 2015) or seeking shelter among groups of conspecifics (Agrillo et al., 2008) animals tend to “go for more.”

More advanced relative numerosity judgments have been explored in laboratory studies with trained animals. When macaques and pigeons were trained to sequentially choose numerosity displays according to ascending numerical values (e.g., 1–2–3), both species showed an ordinal understanding of numerical quantity by transferring their behavior to novel ranges of numerosities (Brannon and Terrace, 1998; Scarf et al., 2011; Scarf and Colombo, 2020). Nevertheless, judging relative numerosity is probably the simplest form of numerical competence because it does not require a representation of the absolute quantity values.

Many classic studies primarily using rodents trained these animals to detect one and the same specific numerosity as a rewarded conditioned stimulus. For instance, rodents were trained to discriminate two specific numbers of sensory signals (Fernandes and Church, 1982; Davis and Albert, 1986) or to produce one specific number of lever presses to receive a reward (Mechner, 1958; Meck and Church, 1983; Çavdaroğlu and Balcı, 2016). However, rodents and many other vertebrates so far have never been trained to flexibly detect any possible absolute numerosity in random trials. Only if animals can flexibly represent any specific numerosity from any other value do they show absolute numerosity representations. Besides humans, only simian primates (*chimpanzees:* Matsuzawa, 1985; Murofushi, 1997; *rhesus macaque:* Cantlon and Brannon, 2007a; Merten and Nieder, 2009) and selected bird species (*parrot:* Pepperberg, 1994; *pigeons:* Xia et al., 2001; *corvids:* Smirnova et al., 2000; Ditz and Nieder, 2016) have been shown to master flexible absolute numerosity judgments. This suggests qualitative differences in numerical intelligence between species.

**FIGURE 1:**
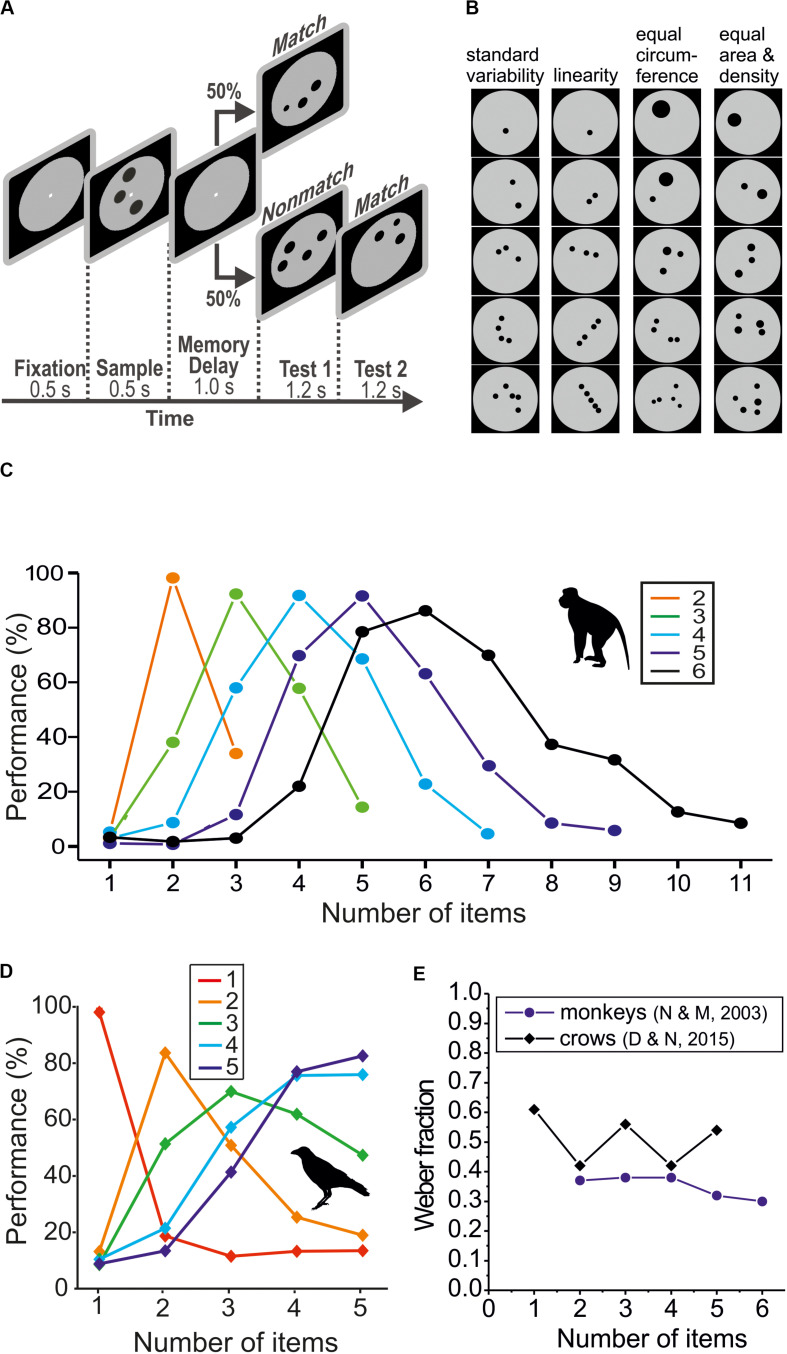
Discrimination performance for simultaneously presented small numerosities. **(A)** Layout of the delayed match-to-numerosity task (DMNT) for dot arrays. **(B)** Example stimulus protocols for numerosity 1–5 that control for different non-numerical parameters. **(C)** Average numerosity performance functions of two rhesus macaques in the DMNT for target numerosity 2–6 (data from Nieder and Miller, 2003). **(D)** Average numerosity performance functions of two carrion crows in the DMNT for target numerosity 1–5 (data from Ditz and Nieder, 2015). **(E)** Weber fractions for small simultaneous-numerosity discriminations of two macaques and two crows. Weber fractions derived from the functions shown in **(C,D)**, respectively.

Absolute numerosity discriminations have been investigated in different vertebrate species using a delayed match-to-numerosity task (DMNT) (Figure 1A; Nieder et al., 2002). In the DMNT, motivated subjects discriminate numerosities that are carefully controlled for non-numerical features for reward (Figure 1B). A typical trial in a visual DMNT begins when a variable target numerosity (the sample) is presented on a screen. The subject has to recognize and then memorize the numerosity over a brief delay period. If the same target numerosity (a match) is shown again in the subsequent test phase, the subject is required to respond. However, if a deviant (smaller or larger) numerosity (a non-match) is presented in the test phase, the subject must withhold responding and wait for the next test stimulus, which always is a match. Match and non-match are presented with equal probability of *p* = 0.5. The accuracy of numerosity discrimination performance is calculated by dividing the number of correct responses by the number of total responses (correct plus erroneous responses) for the match and all non-match test stimuli.

Using a DMNT with virtually identical experimental conditions, detailed psychophysical characterization of absolute numerosity representations have been obtained in humans (Merten and Nieder, 2009), rhesus macaques (Nieder and Miller, 2003; Nieder et al., 2006; Merten and Nieder, 2009; Nieder, 2012), and carrion crows (Ditz and Nieder, 2015, 2016, 2020). These data allow us to characterize the subjective representations of numerosity in detail. When both smaller and larger non-match numerosity displays are presented besides the matching target numerosity, the subjects’ responses give rise to bell-shaped performance functions (or “probability density functions”) (Figure 1C). These performance functions represent the likelihood that any number is perceived as being equal to a specific objective target number (typically located at the center of the function). For instance, two monkeys made most mistakes for non-match numerosity adjacent to the target numerosity; only with increasing numerical distance of the non-match numerosities from the target numerosity did the monkeys err less and less, which resulted in the slopes of the bell-shaped performance functions fading away (Nieder and Miller, 2003). Thus, the performance functions graphically indicate a subject’s subjective numerical representation of objective numbers.

## Quantification of Number Discrimination Accuracy

The finding that absolute numerosity discriminations result in performance distributions of some width clearly shows that the non-symbolic discrimination of numerical quantity is an approximate estimation process. Several psychophysical signatures of non-symbolic number representations can be extracted from these performance functions. First, while similar numerical quantities are difficult to discriminate, discrimination performance systematically improves with increasing difference (or distance) between two quantities; this finding is called “numerical distance effect.” Second, discrimination worsens at the same time with increasing magnitudes so that the numerical distance between numerosities must increase in proportion with the absolute magnitudes to enable discrimination; this phenomenon is called the ‘numerical size effect.’ Both numerical distance and size effects are captured by *Weber’s law*. It states that the just-noticeable difference (“JND,” Δ*I*, or “difference limen”; i.e., the stimulus difference that allows 50% correct discrimination) between two magnitudes divided by the reference magnitude, *I*, is a *constant* (ΔI/I = c) (Weber, 1850). The widths of the resulting performance distributions reflect the numerical distance effect, while the progressive broadening of the functions in proportion to increasing magnitude mirror the numerical size effect (Figure 1C).

In addition, a third signature surfaces on top of Weber’s law: relative to a given reference number, subjects find it easier to discriminate smaller numbers, and more difficult to discriminate larger number (Figure 1C). This effect results in performance functions being mildly asymmetric when plotted on a linear number scale (Figure 1C). This asymmetry of the performance functions is predicted by Fechner’s law which states that the subjective sensation of number, *S*, is proportional to the logarithm of the objective stimulus magnitude, *I* [*S = k log(I)*] (Fechner, 1860). Both Weber’s and Fechner’s laws hold true in psychophysical assessments of numerosity discriminations across species (Nieder and Miller, 2003; Merten and Nieder, 2009; Ditz and Nieder, 2016). Signatures of Weber’s law in numerosity discrimination are a clear sign of an internal “approximate number system” (ANS). The ANS has been found consistently for numerosity judgments in innumerate humans (Gordon, 2004; Pica et al., 2004; Frank et al., 2008) or humans prevented from counting (Whalen et al., 1999; Cordes et al., 2001; Merten and Nieder, 2009), as well as in a multitude of animal species (Nieder, 2020b) from primates (Nieder and Miller, 2003; Cantlon and Brannon, 2006) to bees (Dacke and Srinivasan, 2008; Howard et al., 2018).

**FIGURE 2:**
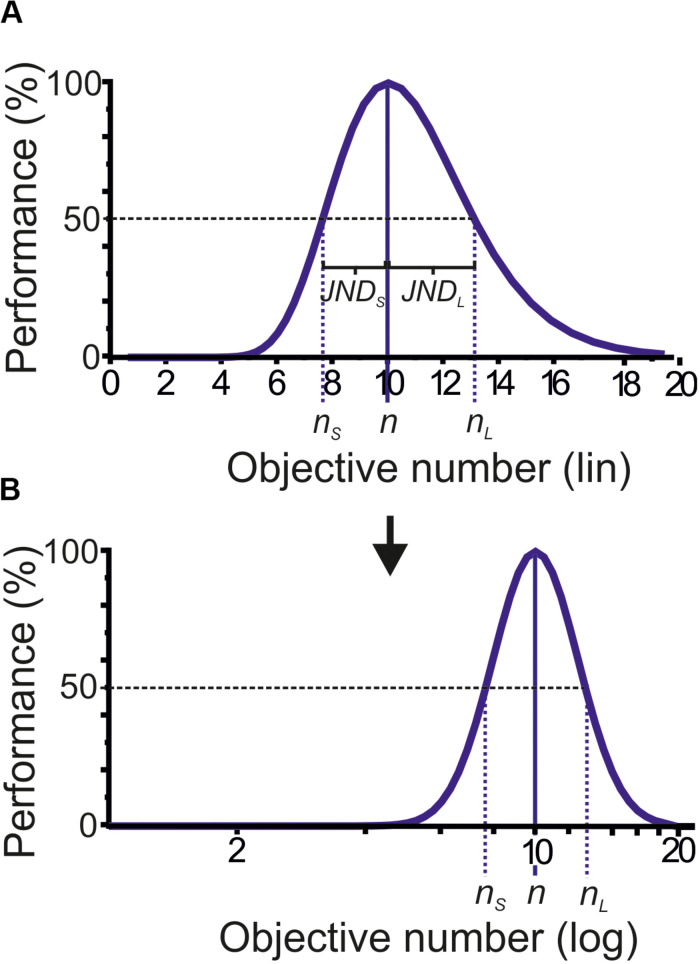
Ideal numerosity performance function. **(A)** Ideal numerosity performance function for target numerosity 10 plotted on a linear number scale (*top graph*). The function shows a steeper slope toward smaller, and a shallower slope toward higher numerosity. As a result, the just-noticeable difference (JND, indicated by dotted colored lines) at which numerosities smaller (*n*_S_) and larger numerosities (*n*_L_) can be discriminated in 50% from the target (*n*) is smaller on the left compared to the right side of the function. **(B)** When the same function is plotted on a logarithmic number scale, the function becomes symmetric and the JNDs are equal on either side of the function (*bottom graph*).

To quantify discrimination accuracy, the Weber fraction is calculated. The Weber fraction expresses how much two stimuli need to differ in magnitude in order for a subject to be able to detect a difference between those two stimuli (i.e., “JND” or “difference limen”). Due to the logarithmic relationship that is stated by Fechner’s law and has been confirmed experimentally for numerosity discriminations in humans, monkeys, and crows (Nieder and Miller, 2003; Merten and Nieder, 2009; Ditz and Nieder, 2016; Piantadosi and Cantlon, 2017) the JND (and thus the Weber fractions) for numerosities smaller and larger than the target numerosity differ (Figure 2A). The JND_S_ (*n*-*n*_S_) for numerosities smaller (*n*_S_) that the target (*n*) is smaller than the JND_L_ (*n*-*n*_*R*_) for numerosities larger (*n*_*R*_) that the target (*n*). Therefore, the left (toward smaller) and right (toward higher numbers) segments of the performance function need to be calculated separately when plotted on a linear number axis (van Oeffelen and Vos, 1982). Thus, the Weber fraction (*W*_S_) for numerosities smaller than the target is

(1) $$W_{\text{S}}=\left(n-n_{\text{S}}\right)/n_{\text{S}}$$

The Weber fraction (W_L_) for numerosities larger than the target is

(2) $$W_{\text{L}}=\left(n_{\text{L}}-n\right)/n$$

To arrive at a single Weber-fraction value for a target numerosity, *W*_S_ and *W*_L_ need to be averaged. Alternatively, the data can be plotted on a logarithmic scale in agreement with Fechner’s law, which renders the JND toward smaller and larger numerosities equal (Figure 2B). The smaller the Weber fraction, the higher is the discrimination accuracy. With the Weber fraction as an objective measure of discriminability, the judgment of absolute numerosities can be compared quantitatively.

## Numerosity Discrimination Accuracy With Simultaneously Presented Items

By far most studies dealing with non-symbolic numerosity representations have employed item arrays as stimuli (i.e., **∴**) (Figure 1A). Numerosity stimuli have to be carefully controlled for non-numerical variables because the number of items is intrinsically correlated with many other features of a physical stimulus. For instance, when the number of dots is increased, usually also the total amount of area covered by all dots and the density of the dots increases. Since primates and birds are sensitive to non-numerical magnitudes (Tudusciuc and Nieder, 2010; Moll and Nieder, 2014) a subject could in fact respond to changing item sizes or density rather than numbers. Unfortunately, it is physically not possible to control for all non-numerical factors simultaneously in a single stimulus display. The best way to tackle the problem of non-numerical cues is to control – unbeknown to the subject – one parameter after the other in separate stimulus configurations (Figure 1B). If a subject abstracts across these parameters and responds equally to these systematically varied numerosity stimuli, it is safe to conclude that the subject responds to number. The application of such control stimuli demonstrated that the subjects indeed responded to the number or items, not to non-numerical factors (Nieder et al., 2002; Merten and Nieder, 2009; Ditz and Nieder, 2015, 2016).

When simultaneously presented items are scattered across space, they can be assessed at one glance. This is evidenced by monkeys responding with similar reaction times to different simultaneously presented numerical values (Nieder and Miller, 2004b; Merten and Nieder, 2009). As an exception to this pattern, animals usually respond faster to very small numerosities 1 and 2 (Merten and Nieder, 2009). In addition, when the number of items in the displays increased, the monkeys showed the same number of eye movements prior to a decision; they did not scan individual items one after the other before responding. Both findings indicate that non-symbolic estimation of number in dot arrays is a parallel process because serial enumeration would require increasing reaction times with increasing numerical values (Mandler and Shebo, 1982). Thus, the simultaneous number estimation constitutes a specific type of enumeration that differs from a counting-like sequential process.

In initial studies, monkeys (Figure 1C) and crows (Figure 1D) were required to discriminate small sample numerosities (usually from 1 to 5) from other small numerosities. The average Weber fraction of two rhesus monkeys for sample numerosities 2–5 was 0.36 (+/− 0.03 std) (Nieder and Miller, 2003) which was significantly smaller than the average Weber fraction of 0.49 (+/− 0.07 std) of two carrion crows for the same numerosity range (Ditz and Nieder, 2015) (*p* < 0.05; one-tailed paired *t*-test; *n* = 4) (Figure 1E). Similar small Weber fractions were obtained for a third monkey (see Figure 3B in Merten and Nieder, 2009). Thus, for small numerosities, macaques discriminate more precisely than crows.

**FIGURE 3:**
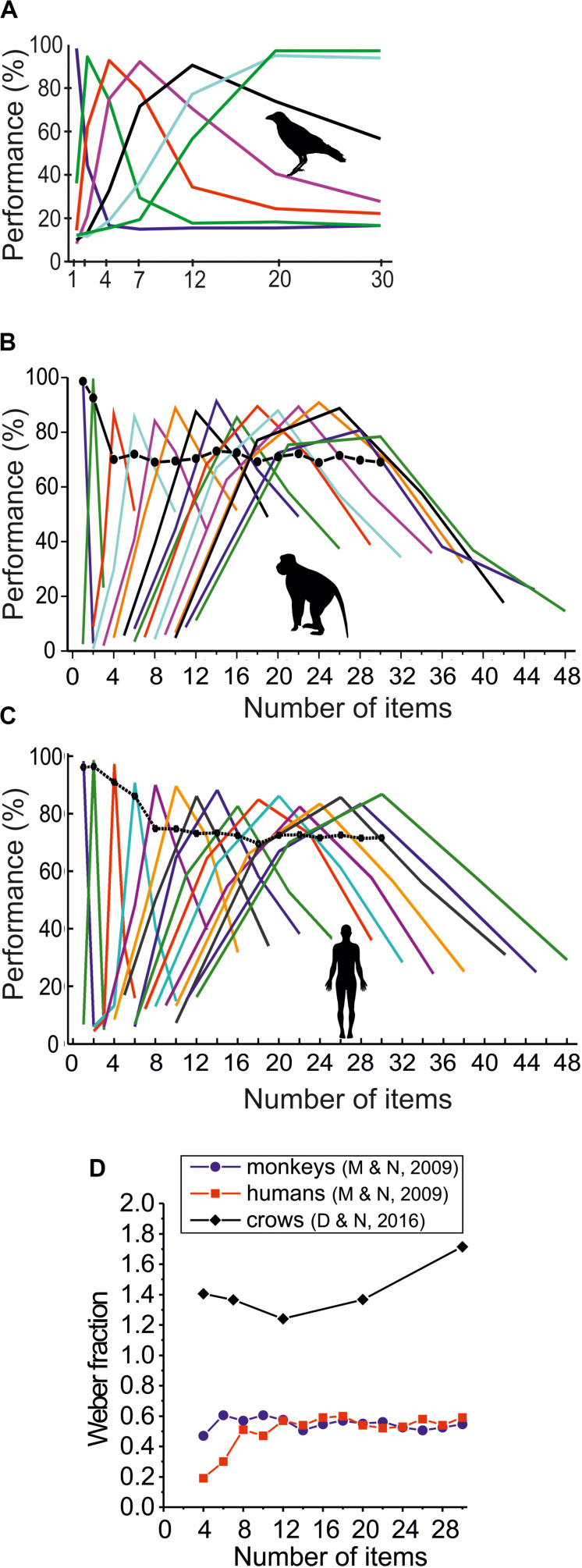
Discrimination performance for simultaneously presented large numerosities. **(A)** Average numerosity performance functions of two carrion crows in the delayed match-to-numerosity task (DMNT) for target numerosity 1–30 (data from Ditz and Nieder, 2016). **(B)** Average numerosity performance functions of two rhesus macaques in the DMNT for target numerosity 1–30 (data from Merten and Nieder, 2009). **(C)** Average numerosity performance functions of 20 humans in the DMNT for target numerosity 1–30 (data from Merten and Nieder, 2009). **(D)** Weber fractions for large simultaneous-numerosity discriminations of two crows, two macaques, and 20 humans. Weber fractions derived from the functions shown in **(A–C)**, respectively.

A similar advantage for primates emerged when larger sample numerosities ranging from 4 to 30 were applied (Figures 3A–C). While the performance of two macaques exhibited an average Weber fraction of 0.55 (+/− 0.04 std) (Merten and Nieder, 2009), crows showed a much higher Weber fraction of 1.42 (+/− 0.18 std) (Ditz and Nieder, 2016) (*p* < 0.05; two-tailed paired *t*-test; *n* = 5) (Figure 3D). The dramatically increased large-numerosity Weber fractions of the same two crows that showed smaller values when tested with small numerosities (Ditz and Nieder, 2015) may partly be explained by much larger numerical distances of the non-match numerosities relative to the sample numerosity. In other words, the crows were not forced to discriminate as precisely as in the previous study in which minimal numerical distances of one between all numerosities were applied (Ditz and Nieder, 2015).

The same study that tested two rhesus macaques also tested 20 adult humans with the same stimuli, apparatus, and protocol (Merten and Nieder, 2009). Due to the rapid presentation of sample and test stimuli, humans were not able to count larger numbers of items symbolically (Figure 3C). Interestingly, humans showed the identical Weber fraction of 0.55 (+/− 0.12) as the two monkeys when non-symbolically discriminating numerosities 4–30 (*p* > 0.05; two-tailed paired *t*-test; *n* = 14). Overall, the data from both small and large numerosity discriminations argue that (human and non-human) primates are more precise when discriminating number in simultaneously presented item arrays.

## Numerosity Discrimination Accuracy With Sequentially Presented Items

**FIGURE 4:**
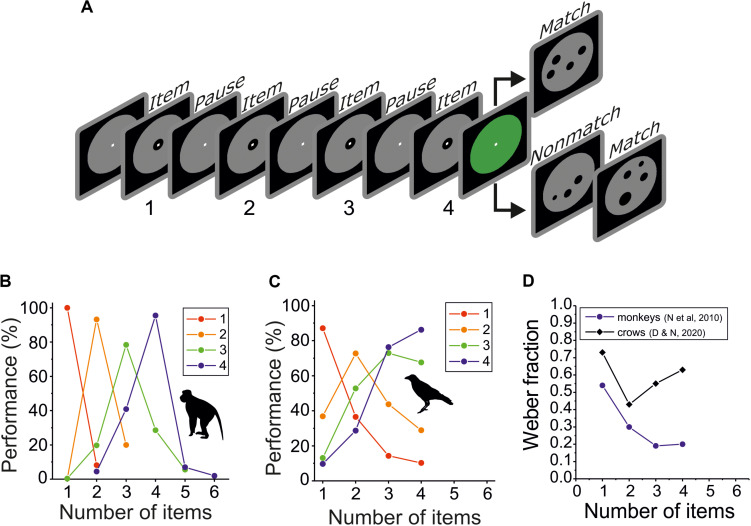
Discrimination performance for sequentially presented numerosity. **(A)** Layout of the delayed match-to-numerosity task (DMNT) for four sequentially presented single dot in the sample period. **(B)** Average numerosity performance functions of two rhesus macaques in the sequential DMNT for target numerosity 1–4 (data from Nieder, 2012). **(C)** Average numerosity performance functions of two carrion crows in the sequential DMNT for target numerosity 1–4 (data from Ditz and Nieder, 2020). **(D)** Weber fractions for small sequential-numerosity discriminations of two macaques and two crows. Weber fractions derived from the functions shown in **(B,C)**, respectively.

The concept of numerosity does not only apply for item arrays, but also for items presented over time (Figure 4A). If items are presented one after the other in a temporal succession (i.e., •- •- •, etc.), they need to be evaluated in sequence. Although only few studies tested sequential enumeration, it is not only more relevant for the auditory and tactile sense, but also more similar to actual counting, which is a sequential process.

Stimuli testing sequential enumeration need to be carefully controlled for temporal variables because it usually takes longer to present more items. The necessary stimulus configurations that control for a variety of temporal factors have been applied in studies with monkeys and crows. They show that the subjects indeed responded to the number of sequentially presented items, and not to temporal factors (Nieder et al., 2006; Nieder, 2012; Ditz and Nieder, 2020).

Detailed performance data for the enumeration of visual sequences of flashed dots are available for two monkeys (Figure 4B; Nieder, 2012) and two crows (Figure 4C; Ditz and Nieder, 2020). With an average Weber fraction of 0.31 (+/− 0.17), the two monkeys showed significantly better accuracy then the two crows with a Weber fraction of 0.59 (+/− 0.13) (*p* < 0.05; two-tailed paired *t*-test; *n* = 4) (Figure 4D). Just as with the simultaneous numerosity protocol, monkeys also outperformed crows in the sequential numerosity protocol.

The monkeys’ performance is reminiscent of the performance of adult humans in non-symbolic sequential enumeration tasks. When human subjects produce target numbers of key presses at rates that made symbolic counting difficult or impossible, or by preventing them from counting by saying “the” at every press, similar precision was reported. In these human studies, the coefficient of variation (CV, the ratio of the standard deviation and mean) was used as a measure of number discriminability (Whalen et al., 1999; Cordes et al., 2001). On average, the CV of humans was around 0.2.

Even though the CV erroneously assumes symmetric performance distributions and is not directly related to the Weber fraction, we calculated the CV for the same monkey (Nieder, 2012) and crow data (Ditz and Nieder, 2020) from Gauss functions fitted to the sequential performance functions. For sequential enumeration, crows had a much larger average CV of 0.39. However, with a value of 0.19, the monkeys demonstrated a discrimination accuracy almost identical to humans. Just as with simultaneous numerosity protocols, the non-symbolic numerosity discrimination accuracy of humans and monkeys also matches for sequential protocols and surpassed those of crows.

## From Behavior to Neurons

The controlled DMNT not only allows a detailed characterization of behavioral numerosity representations, but also offers the opportunity of combining behavioral and brain research. Not only does combining controlled behavior with simultaneous neurophysiological recordings give us a direct way to learn about how the brain gives rise to numerical competence, it also allows us a way to derive more objective signatures of cognitive capabilities at the level of the neural substrate.

The neuronal mechanisms of absolute numerosity representations in the endbrains of the three species show an impressive correspondence. A significant proportion of single neurons in the human medial temporal lobe (Kutter et al., 2018) the monkey frontal and parietal association cortices (Nieder et al., 2002; Nieder and Miller, 2004a) and the avian brain region “nidopallium caudolaterale” (NCL) (Ditz and Nieder, 2015, 2016) are tuned to individual preferred numerosities presented simultaneously in dot arrays. This approximate tuning results in peaked neuronal response functions that resemble behavioral performance functions. Just as the behavioral performance functions, the neuronal tuning functions show all the characteristics of the Weber–Fechner law: neurons best discriminate numerosities that are distant from the preferred numerosity (mirroring the distance effect), the neuronal tuning functions become broader with an increase of the neurons’ preferred numerosity (a reflection of the size effect), and finally the neuronal tuning functions are best described (i.e., symmetric) on a logarithmic number scale. Numerosity tuning functions showing these characteristics were also indirectly derived through functional imaging in humans (Piazza et al., 2004; Nieder, 2004; Jacob and Nieder, 2009; Kersey and Cantlon, 2017).

This argues that the way in which numerosity-selective neurons encode numerical quantity gives rise to the psychophysical characteristics captured by the Weber–Fechner law. Moreover, the quantitative parameters derived from the neuronal tuning functions, such as the widths of the tuning functions, are comparable between monkeys and crows (Nieder and Miller, 2003; Ditz and Nieder, 2015). All these findings argue that primates and crows engage the same ANS when representing absolute numerosity.

In the human literature, it is hotly debated whether the brain represents numerosity separately for simultaneous versus sequential presentation formats, or abstractly and format-independently. The neuronal data from monkeys and crows both argue for a neuronal two-stage process when these two fundamentally different number formats need to be represented. During the sensory presentation stage, the number of sequentially presented items is extracted by one population of numerosity-tuned neurons, whereas the numerosity in dot arrays is represented by another population of numerosity-tuned neurons (Nieder et al., 2006; Ditz and Nieder, 2020). At this sensory stage of number processing, neurons therefore responded format-dependently. However, once the sensory presentation phase had ended, yet another neuronal population represents numerosity format-independently. This third, format-independent population of neurons maintains numerical information in working memory and also predicts performance success (Nieder et al., 2006; Ditz and Nieder, 2020). In summary, sequential and simultaneous number formats engage different and temporally succeeding populations of format-dependent and format-independent numerosity-selective neurons.

Combining the DMNT with electrophysiological recordings not only provided insights into the behavioral relevance of sensory number representations (Viswanathan and Nieder, 2015), but also enables insights into how numerical information is maintained in working memory and further processed according to behavioral principles (rules) (Cantlon and Brannon, 2007b; Bongard and Nieder, 2010; Vallentin et al., 2012; Eiselt and Nieder, 2013; Cantlon et al., 2016). An in-depth treatment of the neuronal correlates of number representations is beyond the scope of this article concerned with psychophysical results but can be found in recent reviews (Nieder, 2016, 2020b).

## Conclusion

In his Null Hypotheses, Macphail (1985) suggests that “*neither quantitative nor qualitative differences among the intellects of non-human vertebrates*” existed. The current analyses show that both the quantitative and qualitative aspect of this hypothesis are violated.

The first, quantitative aspect of Macphail (1985) Null Hypotheses proves to be an untenable assertion. As shown in the current review, the three vertebrate species that master elaborate absolute numerosity judgments systematically differ in their precision. The two primate species (humans and monkeys) consistently showed higher (and surprisingly similar) accuracy when discriminating numerosities in a non-symbolic manner. If quantitative differences emerge already for only three investigated vertebrate species, even more pronounced differences can be expected for a broader range of vertebrate species.

In addition, also the second, qualitative aspect of Macphail (1985) Null Hypotheses proves to be an untenable assertion. This is because abstract and flexible judgments of absolute numerosity have so far only been mastered by humans, simian primates and selected bird species, mammalian and avian species that belong to the most cognitively advanced vertebrate classes. This suggests that species from other vertebrate classes (fish, amphibians, and non-avian reptiles) are not capable of flexible absolute numerosity representations. Of course, one may argue that the blank spots of numeracy in the vertebrate phylogenetic tree will be filled with time and more investigations. After all, fish (DeLong et al., 2017) amphibians (Uller et al., 2003), and non-avian reptiles (Gazzola et al., 2018) show relative numerical competence. In fact, some species of teleost fish show unexpected numerical (Miletto et al., 2020) and cognitive skills (Bloch et al., 2019) suggesting that they may also grasp absolute numerosity judgments. However, I predict that amphibians and non-avian reptiles will never master absolute numerosity tasks because they seem to lack the necessary behavioral flexibility (or intelligence) to solve such abstract tasks.

In sum, and in contrast to Macphail’s (1985) Null Hypotheses, clear quantitative as well as qualitative differences among the numerical intellects of non-human vertebrates exist. In the field of numerical competence, and most likely also across other cognitive competence, Macphail’s Null Hypotheses is untenable.

## Author Contributions

AN conceptualized and wrote the manuscript.

## Conflict of Interest

The authors declare that the research was conducted in the absence of any commercial or financial relationships that could be construed as a potential conflict of interest.

## References

[B1] Agrillo C. Dadda M. Serena G. Bisazza A. (2008). Do fish count? spontaneous discrimination of quantity in female mosquitofish. *Anim. Cogn.* 11 495–503. 10.1007/s10071-008-0140-9 18247068

[B2] Bloch S. Froc C. Pontiggia A. Yamamoto K. (2019). Existence of working memory in teleosts: establishment of the delayed matching-to-sample task in adult zebrafish. *Behav. Brain Res.* 370:111924. 10.1016/j.bbr.2019.111924 31028766

[B3] Bongard S. Nieder A. (2010). Basic mathematical rules are encoded by primate prefrontal cortex neurons. *Proc. Natl. Acad. Sci. U.S.A.* 107 2277–2282. 10.1073/pnas.0909180107 20133872 PMC2836656

[B4] Brannon E. M. Terrace H. S. (1998). Ordering of the numerosities 1 to 9 by monkeys. *Science* 282 746–749. 10.1126/science.282.5389.746 9784133

[B5] Cantlon J. F. Brannon E. M. (2006). Shared system for ordering small and large numbers in monkeys and humans. *Psychol. Sci.* 17 401–406. 10.1111/j.1467-9280.2006.01719.x 16683927

[B6] Cantlon J. F. Brannon E. M. (2007a). Basic math in monkeys and college students. *PLoS Biol.* 5:e328. 10.1371/journal.pbio.0050328 18092890 PMC2140091

[B7] Cantlon J. F. Brannon E. M. (2007b). How much does number matter to a monkey (*Macaca mulatta*)? *J. Exper. Psychol.* 33 32–41. 10.1037/0097-7403.33.1.32 17227193

[B8] Cantlon J. F. Merritt D. J. Brannon E. M. (2016). Monkeys display classic signatures of human symbolic arithmetic. *Anim. Cogn.* 19 405–415. 10.1007/s10071-015-0942-5 26660686 PMC6063318

[B9] Çavdaroğlu B. Balcı F. (2016). Mice can count and optimize count-based decisions. *Psychon. Bull. Rev.* 23 871–876. 10.3758/s13423-015-0957-6 26463617

[B10] Cordes S. Gelman R. Gallistel C. R. Whalen J. (2001). Variability signatures distinguish verbal from nonverbal counting for both large and small numbers. *Psychon. Bull. Rev.* 8 698–707. 10.3758/bf03196206 11848588

[B11] Dacke M. Srinivasan M. V. (2008). Evidence for counting in insects. *Anim. Cogn.* 11 683–689. 10.1007/s10071-008-0159-y 18504627

[B12] Davis H. Albert M. (1986). Numerical discrimination by rats using sequential auditory stimuli. *Anim. Learn. Behav.* 14 57–59. 10.3758/bf03200037

[B13] DeLong C. M. Barbato S. O’Leary T. Wilcox K. T. (2017). Small and large number discrimination in goldfish (*Carassius auratus*) with extensive training. *Behav. Process.* 141 (Pt 2), 172–183. 10.1016/j.beproc.2016.11.011 27890598

[B14] Ditz H. M. Nieder A. (2015). Neurons selective to the number of visual items in the corvid songbird endbrain. *Proc. Natl. Acad. Sci. U.S.A.* 112 7827–7832. 10.1073/pnas.1504245112 26056278 PMC4485087

[B15] Ditz H. M. Nieder A. (2016). Numerosity representations in crows obey the Weber-Fechner law. *Proc. R. Soc. B* 283:20160083. 10.1098/rspb.2016.0083 27009227 PMC4822466

[B16] Ditz H. M. Nieder A. (2020). Format-dependent and format-independent representation of sequential and simultaneous numerosity in the crow endbrain. *Nat. Commun.* 11:686. 10.1038/s41467-020-14519-2 PMC7000399 32019934

[B17] Eiselt A. K. Nieder A. (2013). Representation of abstract quantitative rules applied to spatial and numerical magnitudes in primate prefrontal cortex. *J. Neurosci.* 33 7526–7534. 10.1523/jneurosci.5827-12.2013 23616557 PMC6619563

[B18] Fechner G. T. (1860). *Elemente der Psychophysik*, Vol. 2 Leipzig: Breitkopf & Härtel.

[B19] Fernandes D. M. Church R. M. (1982). Discrimination of the number of sequential events. *Anim. Learn. Behav.* 10 171–176. 10.3758/bf03212266

[B20] Frank M. C. Everett D. L. Fedorenko E. Gibson E. (2008). Number as a cognitive technology: evidence from Pirahã language and cognition. *Cognition* 108 819–824. 10.1016/j.cognition.2008.04.007 18547557

[B21] Gazzola A. Vallortigara G. Pellitteri-Rosa D. (2018). Continuous and discrete quantity discrimination in tortoises. *Biol. Lett.* 14:20180649. 10.1098/rsbl.2018.0649 30958247 PMC6303513

[B22] Gordon P. (2004). Numerical cognition without words: evidence from amazonia. *Science* 306 496–499. 10.1126/science.1094492 15319490

[B23] Howard S. R. Avarguès-Weber A. Garcia J. E. Greentree A. D. Dyer A. G. (2018). Numerical ordering of zero in honey bees. *Science* 360 1124–1126. 10.1126/science.aar4975 29880690

[B24] Jacob S. N. Nieder A. (2009). Tuning to non-symbolic proportions in the human frontoparietal cortex. *Eur. J. Neurosci.* 30 1432–1442. 10.1111/j.1460-9568.2009.06932.x 19788575

[B25] Kersey A. J. Cantlon J. F. (2017). Neural tuning to numerosity relates to perceptual tuning in 3-6-year-old children. *J. Neurosci.* 37 512–522. 10.1523/jneurosci.0065-16.2016 28100735 PMC5242404

[B26] Kutter E. F. Bostroem J. Elger C. E. Mormann F. Nieder A. (2018). Single neurons in the human brain encode numbers. *Neuron* 100 753–761.e4. 10.1016/j.neuron.2018.08.036 30244883

[B27] Macphail E. (1985). Vertebrate intelligence: the null hypothesis. *Philos. Trans. R. Soc. Lond. Ser. B Biol. Sci.* 308 37–51. 10.1098/rstb.1985.0008

[B28] Macphail E. (1987). The comparative psychology of intelligence. *Behav. Brain Sci.* 10 645–695.

[B29] Mandler G. Shebo B. J. (1982). Subitizing: an analysis of its component processes. *J. Exp. Psychol. Gen.* 111 1–22. 10.1037/0096-3445.111.1.1 6460833

[B30] Matsuzawa T. (1985). Use of numbers by a chimpanzee. *Nature* 315 57–59. 10.1038/315057a0 3990808

[B31] Mechner F. (1958). Probability relations within response sequences under ratio reinforcement. *J. Exp. Anal. Behav.* 1 109–121. 10.1901/jeab.1958.1-109 16811206 PMC1403928

[B32] Meck W. H. Church R. M. (1983). A mode control model of counting, and timing processes. *J. Exp. Psychol. Anim. Behav. Process.* 9 320–334. 10.1037/0097-7403.9.3.320 6886634

[B33] Merten K. Nieder A. (2009). Compressed scaling of abstract numerosity representations in adult humans and monkeys. *J. Cogn. Neurosci.* 21 333–346. 10.1162/jocn.2008.21032 18510443

[B34] Miletto P. M. E. Pecunioso A. Dadda M. Agrillo C. (2020). Searching for the critical p of macphail’s null hypothesis: the contribution of numerical abilities of fish. *Front. Psychol.* 11:55. 10.3389/fpsyg.2020.00055 32116895 PMC7025564

[B35] Miller E. K. Nieder A. Freedman D. J. Wallis J. D. (2003). Neural correlates of categories and concepts. *Curr. Opin. Neurobiol.* 13 198–203. 10.1016/s0959-4388(03)00037-0 12744974

[B36] Moll F. W. Nieder A. (2014). The long and the short of it: rule-based relative length discrimination in carrion crows, *Corvus corone*. *Behav. Process.* 107 142–149. 10.1016/j.beproc.2014.08.009 25151937

[B37] Murofushi K. (1997). Numerical matching behavior by a chimpanzee (Pan troglodytes): subitizing and analogue magnitude estimation. *Jpn. Psychol. Res.* 39 140–153. 10.1111/1468-5884.00050

[B38] Nieder A. (2004). The number domain- can we count on parietal cortex? *Neuron* 44 407–409. 10.1016/j.neuron.2004.10.020 15504322

[B39] Nieder A. (2012). Supramodal numerosity selectivity of neurons in primate prefrontal and posterior parietal cortices. *Proc. Natl. Acad. Sci. U.S.A.* 109 11860–11865. 10.1073/pnas.1204580109 22761312 PMC3406836

[B40] Nieder A. (2016). The neuronal code for number. *Nat. Rev. Neurosci.* 17 366–382. 10.1038/nrn.2016.40 27150407

[B41] Nieder A. (2019). *A Brain for Numbers: The Biology of the Number Instinct.* Cambridge, MA: MIT Press.

[B42] Nieder A. (2020a). Neural constraints on human number concepts. *Curr. Opin. Neurobiol.* 60 28–36. 10.1016/j.conb.2019.10.003 31810008

[B43] Nieder A. (2020b). The adaptive value of numerical competence. *Trends Ecol. Evol.* 35 605–617. 10.1016/j.tree.2020.02.009 32521244

[B44] Nieder A. Diester I. Tudusciuc O. (2006). Temporal and spatial enumeration processes in the primate parietal cortex. *Science* 313 1431–1435. 16960005 10.1126/science.1130308

[B45] Nieder A. Freedman D. J. Miller E. K. (2002). Representation of the quantity of visual items in the primate prefrontal cortex. *Science* 297 1708–1711. 10.1126/science.1072493 12215649

[B46] Nieder A. Miller E. K. (2003). Coding of cognitive magnitude: compressed scaling of numerical information in the primate prefrontal cortex. *Neuron* 37 149–157. 12526780 10.1016/s0896-6273(02)01144-3

[B47] Nieder A. Miller E. K. (2004a). A parieto-frontal network for visual numerical information in the monkey. *Proc. Natl. Acad. Sci. U.S.A.* 101 7457–7462. 10.1073/pnas.0402239101 15123797 PMC409940

[B48] Nieder A. Miller E. K. (2004b). Analog numerical representations in rhesus monkeys: evidence for parallel processing. *J. Cogn. Neurosci.* 16 889–901. 10.1162/089892904970807 15200715

[B49] Pepperberg I. M. (1994). Numerical competence in an African grey parrot (*Psittacus erithacus*). *J. Comp. Psych.* 108 36–44. 10.1037/0735-7036.108.1.36

[B50] Piantadosi S. T. Cantlon J. F. (2017). True numerical cognition in the wild. *Psychol. Sci.* 28 462–469. 10.1177/0956797616686862 28406373 PMC5407312

[B51] Piazza M. Izard V. Pinel P. Le Bihan D. Dehaene S. (2004). Tuning curves for approximate numerosity in the human intraparietal sulcus. *Neuron* 44 547–555. 10.1016/j.neuron.2004.10.014 15504333

[B52] Pica P. Lemer C. Izard V. Dehaene S. (2004). Exact and approximate arithmetic in an Amazonian indigene group. *Science* 306 499–503. 10.1126/science.1102085 15486303

[B53] Scarf D. Colombo M. (2020). Columban simulation project 2.0: numerical competence and orthographic processing in pigeons and primates. *Front. Psychol.* 10:3017. 10.3389/fpsyg.2019.03017 32038392 PMC6988827

[B54] Scarf D. Hayne H. Colombo M. (2011). Pigeons on par with primates in numerical competence. *Science* 334:1664. 10.1126/science.1213357 22194568

[B55] Smirnova A. A. Lazareva O. F. Zorina Z. A. (2000). Use of number by crows: investigation by matching and oddity learning. *J. Exp. Anal. Behav.* 73 163–176. 10.1901/jeab.2000.73-163 10784007 PMC1284769

[B56] Stancher G. Rugani R. Regolin L. Vallortigara G. (2015). Numerical discrimination by frogs (*Bombina orientalis*). *Anim. Cogn.* 18 219–229. 10.1007/s10071-014-0791-7 25108417

[B57] Tudusciuc O. Nieder A. (2010). Comparison of length judgments and the Müller-Lyer illusion in monkeys and humans. *Exp. Brain Res.* 207 221–231. 10.1007/s00221-010-2452-7 20972775

[B58] Uller C. Jaeger R. Guidry G. Martin C. (2003). Salamanders (*Plethodon cinereus*) go for more: rudiments of number in an amphibian. *Anim. Cogn.* 6 105–112. 10.1007/s10071-003-0167-x 12709845

[B59] Vallentin D. Bongard S. Nieder A. (2012). Numerical rule coding in the prefrontal, premotor, and posterior parietal cortices of macaques. *J. Neurosci.* 32 6621–6630. 10.1523/jneurosci.5071-11.2012 22573684 PMC6621106

[B60] van Oeffelen M. P. Vos P. G. (1982). A probabilistic model for the discrimination of visual number. *Percept. Psychophys.* 32 163–170. 10.3758/bf03204275 7145586

[B61] Viswanathan P. Nieder A. (2015). Differential impact of behavioral relevance on quantity coding in primate frontal and parietal neurons. *Curr. Biol.* 25 1259–1269. 10.1016/j.cub.2015.03.025 25913409

[B62] Weber E. H. (1850). “Der tastsinn und das gemeingefühl,” in *Handwörterbuch der Physiologie Part 2*, Vol. 3 ed. Wagner R. (Nicosia: TP Verone Publishing), 481–588.

[B63] Whalen J. Gallistel C. R. Gelman R. (1999). Nonverbal counting in humans: the psychophysics of number representation. *Psychol. Sci.* 10 130–137. 10.1111/1467-9280.00120

[B64] Xia L. Emmerton J. Siemann M. Delius J. D. (2001). Pigeons (*Columba livia*) learn to link numerosities with symbols. *J. Compar. Psychol.* 115 83–91. 10.1037/0735-7036.115.1.83 11334222

